# Response Letter to: Correspondence regarding the article by Murugan et al on Precision net ultrafiltration dosing in continuous kidney replacement therapy: a practical approach

**DOI:** 10.1186/s40635-023-00588-2

**Published:** 2024-02-20

**Authors:** Raghavan Murugan, Kianoush Kashani, Paul M. Palevsky

**Affiliations:** 1 The Program for Critical Care Nephrology, Department of Critical Care Medicine, University of Pittsburgh School of Medicine, 600 Scaife Hall, 3550 Terrace Street, Pittsburgh, PA 15213 USA; 2 The Center for Clinical Research, Investigation, and Systems Modeling of Acute Illness (CRISMA), Department of Critical Care Medicine, University of Pittsburgh School of Medicine, Pittsburgh, PA USA; 3 Division of Nephrology and Hypertension, Division of Pulmonary and Critical Care Medicine, Department of Medicine, Mayo Clinic, Rochester, MN USA; 4 Renal and Electrolyte Division, Department of Medicine, University of Pittsburgh School of Medicine, Pittsburgh, PA USA; 5 Kidney Medicine Section, Veterans Affairs Pittsburgh Healthcare System, Pittsburgh, PA USA

**To the Editor,**

We read with interest the letter by Bellomo and colleagues in response to our recent article entitled “Precision net ultrafiltration dosing in continuous kidney replacement therapy: a practical approach” [1]. The authors state that we have misplaced the term continuous kidney replacement therapy (CKRT) instead of continuous renal replacement therapy. While we support the use of interchangeable terms such as “renal” and “kidney”, the use of the term “kidney” is more precise and aligns with the Kidney Disease Improving Global Outcome nomenclature [2]. Moreover, several recent publications have used the term CKRT [3–6].

The authors also state that we have subverted the net ultrafiltration (UF_NET_) rate definition we have previously proposed in several publications. Our current publication aims to expand the existing UF_NET_ rate definition by accounting for all intravenous/replacement/dialysate fluids given simultaneously to the patient, not just those administered within the dialysis machine. This is crucial because, as stated in our manuscript, net fluid removal is a form of controlled hypovolemia that leads to cardiovascular stress in the patient. Ignoring fluids administered outside the dialysis machine in the UF_NET_ rate calculation would overestimate the actual UF_NET_ rate dosing and the cardiovascular stress encountered by the patient during net fluid removal. In the recent consensus nomenclature published by the Acute Disease Quality Initiative [7], UF_NET_ in Table 1 is defined as “*Volume of fluid removed from the patient subtracted by volume of fluid infused to patient during a dialysis session*”. Thus, in our calculation of the UF_NET_ rate, we subtracted the volume of fluids simultaneously infused into the patient during a dialysis session in addition to the dialysate and replacement fluids (which can also be given outside the dialysis machine) to determine the precise UF_NET_ rate. This method is more precise in determining the UF_NET_ rate and is currently used in a clinical trial comparing alternative UF_NET_ rate strategies for precision UF_NET_ dosing [8].

The authors also state that we did not consider the complex nature of fluid balance in critically ill patients, such as urinary and drain output, gastrointestinal losses, or nutritional input. We agree that fluid balance is complex. Therefore, we proposed including other fluids in the UF_NET_ rate calculation and provided a supplemental UF_NET_ rate calculator that accounts for various input fluids and output fluid losses. Specifically, our manuscript (Step 3) states, “*Enteral and oral feedings and gastrointestinal and drain losses can also be included in determining the precise UF*_*NET*_* rate*.” We acknowledge that the above UF_NET_ rate determination and dosing method is new and hypothesis-generating, hence our publication under this journal’s “Hypothesis articles” section. We believe our method offers significant potential for improving UF_NET_ dosing accuracy.

## Data Availability

Not applicable.
